# Carcinosarcoma of the Lesser Omentum: A Unique Case Report and Literature Review

**DOI:** 10.1097/MD.0000000000003246

**Published:** 2016-04-08

**Authors:** Bei Wang, Kui-Wu Ren, Yun-Chuan Yang, Da-Long Wan, Xue-Jie Li, Zheng-Long Zhai, Le-Le Zhang, Shu-Sen Zheng

**Affiliations:** From the Laboratory of Combined Multi-organ Transplantation, Ministry of Public Health, Key Laboratory of Organ Transplantation, Division of Hepatobiliary and Pancreatic Surgery (BW, K-WR, Y-CY, D-LW, Z-LZ, L-LZ, S-SZ); and Division of Pathology, First Affiliated Hospital, School of Medicine, Zhejiang University, Hangzhou, Zhejiang Province, China (X-JL).

## Abstract

Carcinosarcoma is a rare tumor consisting of epithelial and mesenchymal components, both of which are histologically malignant. It usually runs an aggressive clinical course, with higher metastatic potential than other kinds of carcinomas or sarcomas.

Here, we present an extremely uncommon case of carcinosarcoma occurred in the lesser omental bursa in a 65-year-old Chinese man. Metastasis was observed 2 months after operation and disappeared completely after chemotherapy. Until now, 3 years after surgery, the patient is still alive without any signs or symptoms of recurrence.

To our knowledge, this is the first case of carcinosarcoma originated from lesser omentum. Surgical resection and the ifosfamide-based combination chemotherapy may be effective to carcinosarcoma in the lesser omentum.

## INTRODUCTION

Carcinosarcoma is defined as a rare neoplasm characterized by showing both carcinomatous and sarcomatous components. This rare tumor can locate in various organs, including gastrointestinal tract, liver, breast, especially the uterus, and female reproductive system.^1^ However, carcinosarcoma located in the lesser omentum has not been reported.

The tumor usually runs an aggressive clinical course, with higher metastatic potential than other kinds of carcinomas or sarcomas, and the exact etiology of the tumor remains unknown yet.^2^ It is reported that the risk factors of uterine carcinosarcoma include estrogen, nulliparity, tamoxifen, and pelvic radiation. However, the risk factors and etiology for the omentum carcinosarcoma remains a mystery because of its extremely low incidence. It is still not clearly understood of the histogenesis of carcinosarcoma, with 3 main hypotheses having been proposed: collision, combination, and conversion theories.^2,3^ Complete surgical resection of primary lesions with wide margins and suitable dissection of lymphnodes is usually a reasonable therapy. For uterine carcinosarcoma—the most frequently occurring carcinosarcoma—combination therapies including postoperative chemotherapy and radiation therapy are also indicated to be effective.^4–7^

We presented an extremely uncommon case of carcinosarcoma originated from the lesser omentum. Solitary liver metastasis was detected 2 months after the operation and disappeared completely after adjunctive chemotherapy. At present, the patient is still alive with a good quality of life.

## CASE PRESENTATION

**FIGURE 1:**
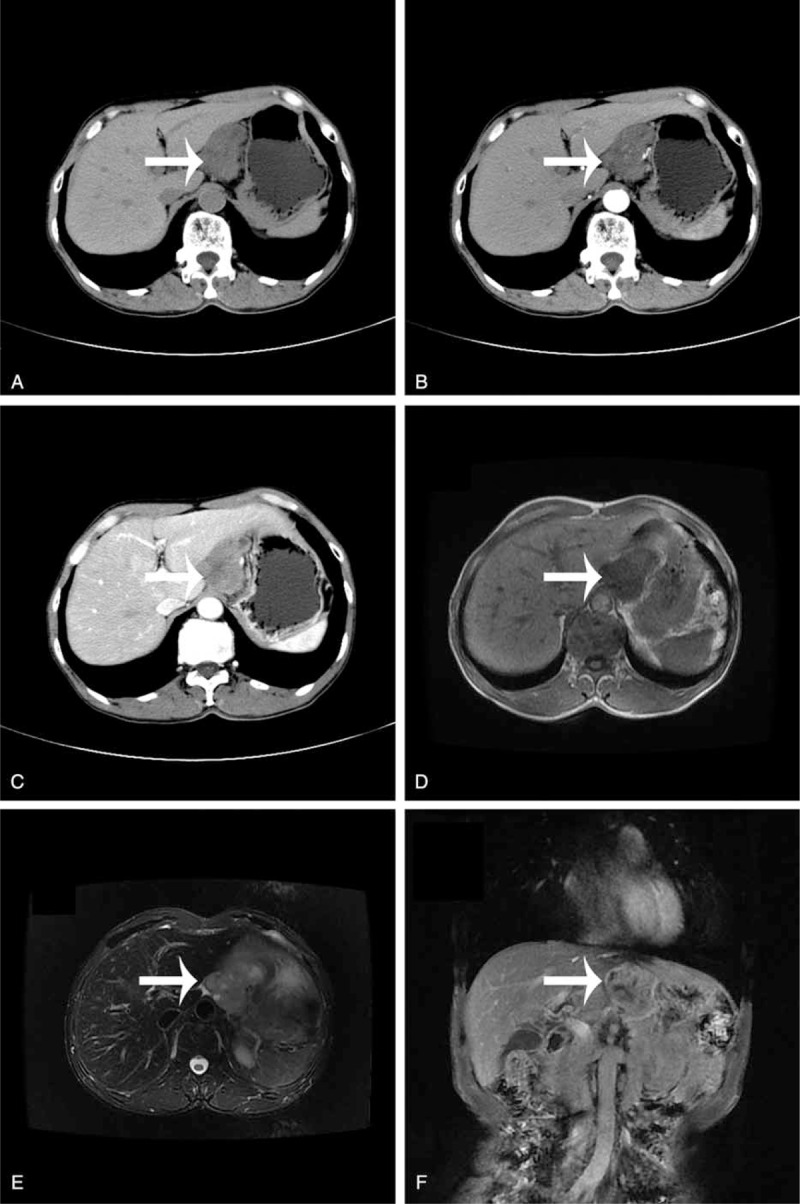
The presence of the carcinosarcoma in the CT and MRI before the peration. A, The CT showed a 5.4 × 4.1 cm^2^ irregular soft tissue mass (white arrow) in the space between the liver and stomach. B, No obvious enhancement (white arrow) was showed in the enhanced abdominal CT scan. C, Slight enhancement (white arrow) was observed in the venous phase. D, The tumor (white arrow) was high signal in T1-weighted MRI. E, T2-weighted imaging revealed no enhancement of the tumor (white arrow). F, The tumor (white arrow) was located in the lesser omentum bursa. CT = computed tomography, MRI = magnetic resonance imaging.

**FIGURE 2:**
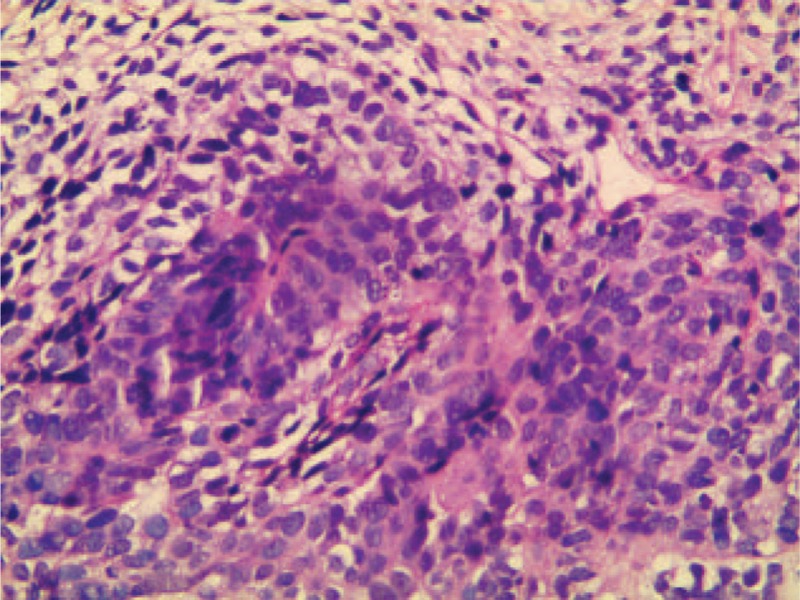
Histologic images of EUS (hematoxylin and eosin [H&E] staining; 200×).

A 65-year-old Chinese man was admitted to our hospital with a complaint of upper abdominal distention. There was no positive sign on physical examination. Tumor markers including carcino-embryonic antigen, carbohydrate atigen 19-9, alpha fetoprotein, and prostate specific antigen were normal. Abdominal enhanced computed tomography (CT) scan revealed a 5.4 × 4.1 cm^2^ irregular soft tissue mass in the space between the liver and stomach (Figure 1A). In the arterial and venous phase, the mass enhanced gradually (Figure 1B and C). The magnetic resonance imaging (MRI) showed the mass had long T1 and T2 signal, and the diffusion-weighted imaging (DWI) revealed a high signal of the mass (Figure 1D–F). Histopathological examination of endoscopic ultrasonography (EUS)-guided fine needle biopsy revealed malignant tumor cells (Figure 2). No evidence of distant metastasis was identified. Complete tumor resection and lymphadenectomy were performed. The protocol for the surgical resection was decided together by the patient and a multidisciplinary team from different departments including hepatobiliary surgery, radiology, and chemotherapy.

**FIGURE 3:**
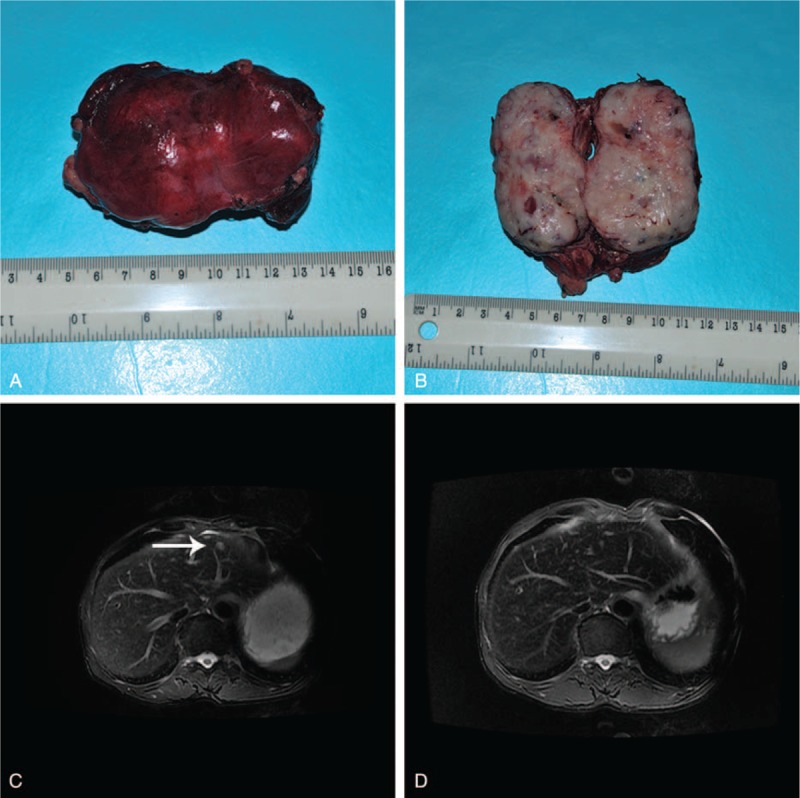
The tumor and MRI after the surgery. A, The tumor measured about 9.0 × 5.0 × 4.5 cm^3^ with intact capsule. B, Inside the tumor, there was “fish-meat” like tissue with necrosis. C, Two months after the operation, the MR images showed a 1 cm metastasis tumor (white arrow) in the left liver. D, After the second chemotherapy, the metastasis tumor disappeared from the left liver. MRI = magnetic resonance imaging.

Grossly, the tumor was situated in the lesser omental bursa and measured 9.0 × 5.0 × 4.5 cm^3^ (Figure 3A). The inside of the tumor presented grayish-white color with necrosis (Figure 3B). Frozen section during the operation revealed that it was a malignant tumor with epithelioid and mesenchymal components.

**FIGURE 4:**
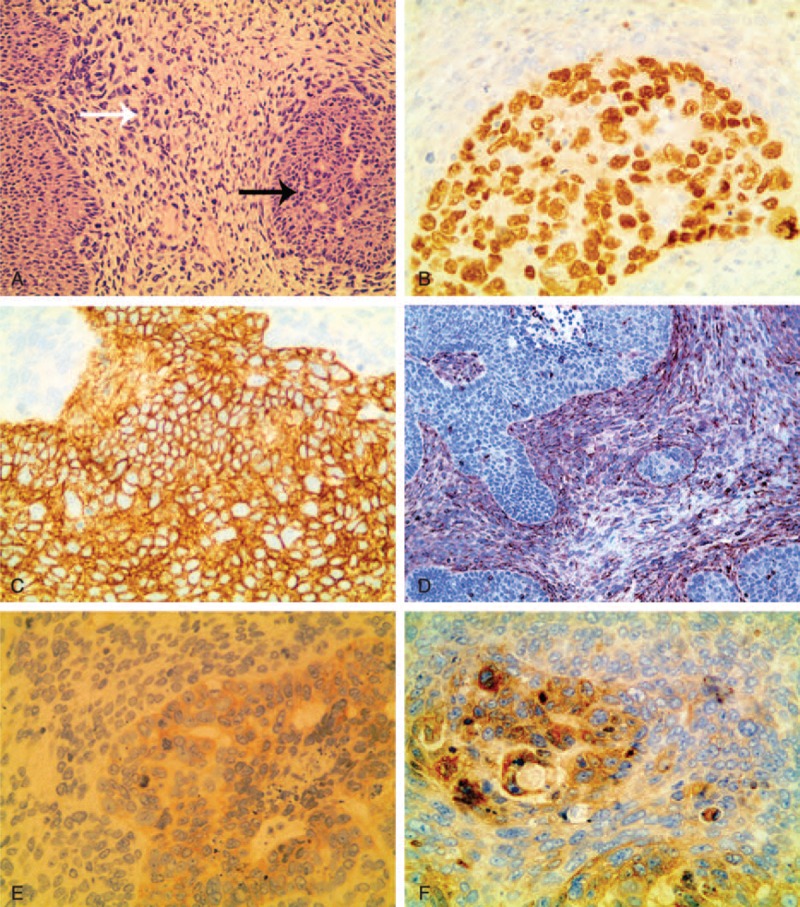
The pathology and histological study of the carcinosarcoma. A, H&E staining (200×) showed that the tumor tissue consisted of 2 well distinguished types of cells: carcinoma cells (black arrow) and sarcoma cells (white arrow). Immunohistochemistry (200×) revealed that tumor cells were positive for p63 (B), CK (C), and VIM (D). Some tumor cells were positive for CD99 (E) and Bcl-2 (F). CK = pan-cytokeratin, H&E = hematoxylin and eosin, VIM = vimentin.

Histologically, the tumor showed biphasic differentiating. The first component was epithelium cells that arranged like cancer nests. Meanwhile, the second component revealed a sarcomatous growth pattern (Figure 4A). Immunohistological study indentified the tumor components by specific labeling techniques. Final diagnosis of carcinosarcoma was confirmed by hematoxylin and eosin (H&E) staining and immunohistochemical analysis showing positive p63, pan-cytokeratin (CK), vimentin (VIM), CD99, and Bcl-2 (Figure 4B–F). Postoperation diagnosis of the patient was primary carcinosarcoma of the lesser omentum without lymph node metastasis.

Two months after the operation, the MRI showed a 1 cm mass in the left liver (Figure 3C) with enlarged lymph nodes in the retroperitoneal area; metastasis was considered. The patient refused a second operation and instead received chemotherapy. The chemotherapeutic protocol (epirubicin 28 mg continuous intravenous infusion 24 hours d1–3 and ifosfemide 2 g d1–3, q3w) was scheduled as 1 time per month and lasted for 6 months till the end. After the chemotherapy regimen, the hepatic MRI showed that the left liver metastases disappeared completely (Figure 3D). Three months later, a hepatic MRI showed no recurrence in the patient's liver. The patient is still alive without any signs of recurrence during 3 years of follow-up.

## DISCUSSION

Carcinosarcoma is a malignant tumor composed of mesenchymal and epithelial components.^8^ This rare tumor locates in various organs, including gastrointestinal tract, liver, breast, especially the uterus, and female reproductive system.^1^ As far as we know, our case is the unique documented case of carcinosarcoma apparently arising from omentum. It is still not clearly understood of the histogenesis of carcinosarcoma, with 3 main hypotheses having been proposed: collision, combination, and conversion theories.^2,3^ The collision theory suggests that the epithelial cells and mesenchymal cells happen to occur and fuse at the common border and give the impression of a single mixed tumor.^9^ The combination theory suggests that both the epithelial and mesenchymal components are derived from a common stem cell to form a single tumor.^10^ The conversion theory is that epithelial cells transformed into malignant cells that give rise to a carcinoma. Also, cells in the carcinoma further undergo metaplastic transformation to give rise to a sarcoma.^11,12^ With the emergence of more and more molecular and genetic data, the conversion theory is being accepted by more scholars.^13^

The lesser omentum is a double layer of the peritoneum that connects the lesser curvature of the stomach and the first part of the duodenum to the porta hepatis. The common tumor of the omentum includes lipoma, liposarcoma, gastrointestinal stromal tumor, and mesothelioma.^14^ However, primary carcinosarcomas originating from the omentum are very rare. Tumors of omentum can often occur insidiously without obvious signs or symptoms, until they grow large enough to compress adjacent organs. The symptoms present as abdominal discomfort, abdominal mass, and abdominal distention. The preoperation diagnosis of omental tumor mainly depends on ultrasound, CT, and MRI images. However, the final diagnosis relies on immunohistological analysis.^15^

Surgical resection is still the main treatment method of omental carcinosarcoma.^8^ However, the effectiveness of surgery and chemotherapy has not been proved for the lack of enough omental carcinosarcoma cases. For uterine carcinosarcoma—the most common carcinosarcomas—R0 resection is usually a reasonable therapy. Combination therapies including postoperative chemotherapy and radiation therapy are also indicated to be effective to reduce the local recurrence of uterine carcinosarcoma, though it may not improve overall survival.^16–19^ Ifosfamide, cisplatin, and paclitaxel have been tried out to be effective against the uterine carcinosarcoma.^10,20^ R0 resection was undertaken in our patient and metastasis was revealed in the left liver about 2 months after the operation. The patient accepted epirubicin and ifosfemide chemotherapy regimen and the metastasis disappeared after the chemotherapy. He felt well and had an uneventful recovery 3 years after the chemotherapy.

## CONCLUSIONS

The lesser omental carcinosarcoma is an extremely rare tumor with high malignancy and aggressive invasiveness that has never been reported. The tumor can metastasize to the adjacent organs. Surgical resection associated with a combination of chemotherapy with ifosfamide and epirubicin may be effective for the tumor.
